# Seroprevalence of Hepatitis-E infection in healthy blood donors in an endemic country

**DOI:** 10.12669/pjms.42.3.11545

**Published:** 2026-03

**Authors:** Lubna Kamani, Adeel Rahat, Sana Anwar

**Affiliations:** 1 Lubna Kamani, FCPS, MRCP(UK), FRCP(London) Professor & Director GI Residency Program, Gastroenterology Department, 3rd Floor, Wajid Ali Shah Building, Liaquat National Hospital, National Stadium Road, Karachi, Pakistan; 2 Adeel Rahat, FCPS Specialist Gastroenterology, King Salman Hospital, Aishah Bint Abi Bakr, Al Uraija Al Wusta, Riyadh 12769, Kingdom of Saudi Arabia; 3 Sana Anwar, FCPS Professor, Department of Microbiology, 3rd Floor, Wajid Ali Shah Building, Liaquat National Hospital, National Stadium Road, Karachi, Pakistan

**Keywords:** Blood donor, Hepatitis E, Seroprevalence, Transfusion-dependent transmission, Pakistan, HEV

## Abstract

**Objective:**

To determine the frequency of Hepatitis-E prevalence among blood donors at a tertiary care hospital in Karachi.

**Methodology:**

A cross-sectional study was conducted among healthy blood donors at the blood donation facility of Liaquat National Hospital (LNH) by Departments of Gastroenterology, Hepatology and Microbiology from October 2020 to January 2022. The blood samples were screened for Hepatitis E virus (HEV) using anti-HEV antibodies (IgG and IgM). Those who tested positive for anti-HEV IgM were tested for HEV-RNA by real-time reverse transcription polymerase chain (PCR).

**Results:**

A total of 515 individuals were screened. The mean age was 30.08±7.56 years, and all were males. The mean ALT values recorded were 42.8447±32. Anti HEV IgG was positive in 366 individuals (71%). Anti HEV IgM was positive in 31 (6%) individuals, but HEV - RNA PCR was negative in all IgM+ individuals.

**Conclusion:**

The high seroprevalence of Anti HEV IgM among healthy blood donors emphasizes the need for robust measures of routine screening for HEV of all blood products in endemic countries to prevent transfusion dependent transmission with special consideration when the recipients are either immunocompromised or pregnant.

## INTRODUCTION

Hepatitis-E Virus (HEV) is a key health concern worldwide, and so is its endemicity in Pakistan. It is one of the prevalent factors causing acute hepatitis.^1^ Infections with HEV follow a particular pathology with an incubation period of 2-6 weeks pursued by a symptomatic phase consisting of one or more symptoms like jaundice, vomiting, abdominal pain, hepatomegaly, etc. Following the commencement of the disease, there is a 2-6 weeks window for IgM detection prior to seroconversion. At this time, rising alanine aminotransferase (ALT) activity can be seen. IgG often takes longer than IgM to manifest, but it can persist for many years. Early in the illness, the levels of HEV RNA and HEV capsid antigen peak and continue for about four weeks.^2^

HEV is found to be self-limiting in healthy individuals but can be associated with significant consequences leading to mortality in pregnant females and immunosuppressed individuals.^3^,^4^ Chronic HEV, particularly in immunocompromised patients and pregnancy, can lead to severe liver disease and fulminant hepatic failure. Obstetrical complications are also more prevalent in patients with HEV infection when compared to non-HEV infected pregnant females.^5^ Vertical transmission is also a possibility which can cause further complications for the newborn, leading to perinatal mortality.^4^ A study conducted by Kamani and colleagues reported an incidence of HEV of 18% in pregnant women.^6^ Complications such as fulminant hepatic failure were reported in about 20.9% with a mortality rate of 1.4%.^6^

Pakistan has also registered HEV vaccine, but as per the WHO position paper, the decision to implement the vaccination regarding HEV has been devised by the local respective authorities.^7^ A new paradigm for the increased prevalence of HEV is found to be blood transfusions.^8^ Blood transfusion related HEV infection was first reported in 2004 in Japan; then it started to escalate and was reported in multiple countries.^8^ Blood transfusions remain the mainstay of treatment for severely anemic patients particularly with pregnancy and major surgeries; post-operative blood transfusion is also among the first line of agents in elective surgeries.^9^

In Pakistan, National blood policy was formulated which focused on legal framework and safety of blood transfusion recipients, but it is also limited to screening for Hepatitis B and C and routine screening of blood products for HEV has not been implemented yet.^10^ Despite increasing evidence of HEV transmission through blood products and its potential for severe complications in vulnerable populations, there remains a significant gap in the understanding of its prevalence among blood donors in Pakistan. This gap poses a potential public health risk, especially considering the high demand for transfusions in pregnant and surgical patients. Assessing the frequency of HEV among blood donors is essential to inform future screening policies, reduce the risk of transfusion-transmitted infections, and safeguard at-risk populations. The objective of the study was to determine the frequency of hepatitis E prevalence among blood donors at a tertiary care hospital in Karachi. To the best of the authors’ knowledge, this is the first study conducted to address the unaccounted prevalence of HEV to the donors.

## METHODOLOGY

This cross-sectional study was conducted among healthy blood donors at the blood donation facility of Liaquat National Hospital (LNH) at Departments of Gastroenterology, hepatology and Microbiology from October 2020 to January 2022.

### Ethical Approval:

The study was approved by Ethical Review Committee (0549-2020-LNH-ERC, of Liaquat National Hospital; Dated: October 1, 2020). Individuals were recruited in accordance with the approval, briefed on the process, and consent forms were obtained.

The study population comprised all patients presenting to the participating hospitals/clinics meeting the eligibility criteria during the study period. Consecutive eligible patients presenting during the study period were enrolled until the target sample size was achieved. The sample size was calculated to be 500 participants using a formula with finite population correction using prevalence as reported by Butt AS et al., keeping confidence interval of 95% and a margin of error to be 2.68%.^10^-^13^

### Inclusion Criteria:

Adults aged ≥18 years were included, consecutive patients who came for blood donation and provided informed consent at Liaquat National Blood bank. The inclusion criteria of blood donation was fulfilled as per the institution practice as well. It was also considered that the subject must be living in Pakistan for more than six months.

### Exclusion criteria:

Included aged <18, patients who refused to give consent, recent history of viral/febrile illness within six weeks, history of viral hepatitis or EBV/CMV to avoid false positivity, autoimmune diseases or hepatotoxic/herbal drugs, pregnant females, hematological disorder or malignant disease.^14^

Initially, the blood samples from all the participants were screened for the presence of anti HEV antibodies. Blood samples received in the Microbiology Laboratory were centrifuged at 4000 rpm for 10 minutes to separate the serum. The HEV IgG and IgM were performed from the serum using NovaLisa Hepatitis E Virus (HEV) IgG & IgM ELISA kits. ELISA was performed using the ETI-MAX 3000 which is a fully automated microtiter plate analyzer performing the complete sample processing (sample pre-dilutions, sample and reagent dispensing, incubations, wash processes, plate transports) as well as the photometric measurement and evaluation following the manufacturer’s instruction. The results were interpreted according to the cutoffs provided by the kit manufacturer.

Individuals with a positive anti-HEV IgM were tested for HEV- RNA PCR using the Fast Track Diagnostics (FTD) Hepatitis E virus Research Use Only (RUO) kit. The viral RNA was isolated using a Qiagen viral RNA/DNA extraction kit (detection limit 500 copies). The isolated target RNA was reverse transcribed into cDNA during the reverse transcription step. The targeted DNA molecules were then amplified simultaneously in the same tube by real-time PCR. The presence of specific sequences was detected by an increase in fluorescence observed from the relevant dual-labeled probe. Demographic details, detailed medical history, and concomitant medications data were obtained using patient history forms and questionnaire. Funding source for HEV PCR detection kits was arranged by Ferzosons Laboratories.

Data was recorded in Case Report Form (CRF) prepared for this study. The CRF comprised of required information in various domains such as demographic information (age, gender, marital status, education, occupation, height, weight), vital signs (blood pressure, heart rate, temperature), & lifestyle factors such as smoking, alcohol use, and drug abuse. It also included confirmation of eligibility, medical history (such as liver disease, glucose intolerance, autoimmune diseases, and HIV co-infection), and concomitant medications. The laboratory results were recorded for pre-donation testing (HBsAg, anti-HCV, HIV I & II, malaria, syphilis) and Hepatitis E-specific tests (anti-HEV IgG, anti-HEV IgM, HEV PCR, and ALT levels). Each signed CRF was checked and signed by the principal investigator for completeness and accuracy before entering data. SPSS version 22 was used to analyze the data which is represented as mean and standard deviation (SD). Categorical variables are presented as frequencies and percentages. T-tests were applied to check for any mean difference between positivity for IgG and IgM for HEV and ALT value > 41 and BMI>25 was considered above the normal level.

## RESULTS

A total of 515 individuals through convenience sampling were enrolled. All the participants were male (100%). The mean age was 30.08 ± 7.56 years [R: 18 - 54 years]. ALT <41 U/L was considered normal for males as per biochemistry lab reference. The mean ALT value was 42.85 ± 32.30U/L. Anti HEV IgG was positive in 366 individuals (71%), whereas Anti HEV IgM was positive in 31 (6%) participants.

Mean ALT and BMI were compared between HEV positive and negative groups ALT value > 41 and BMI>25, which is considered above the normal range. A total of 195 individuals had ALT>41 with a mean BMI of 28.49 ± 5.61 kg/m^2^. Out of these, 141 were positive for Anti HEV IgG (*p*=0.23), their mean ALT was 70.7 ± 41.04 U/L, and seven were positive for Anti HEV IgM (*p*=0.02), their mean ALT was 110.85 ±129.97 U/L. Individuals with ALT values <41 were 320 with a mean BMI of 25.61 ± 5.13. Individuals with both BMI and ALT above the considered normal values were 140 with mean ALT 68.97 ± 41.58 U/L and the mean BMI to be 30.77 ± 4.92 which showed significant difference of means with the incidence of HEV IgM positivity (*p*=0.05).

Participants who tested positive for HBsAg, Anti- HCV Ab, Syphilis and HIV were 11 (2.1%), nine (1.7%), nine (1.7%) and zero (0%) respectively. The PCR results showed no expression for any of the samples.

Characteristics of HEV in patients with ALT >41 U/L with respect to BMI are shown in Table-I while Table-II and III define age wise incidence of IgM and IgG seroprevalence among the studied population.

**Table-I:** Characteristics of HEV in patients with ALT >41 U/L.

Characteristic		Observed ALT Value (Mean ± SD)	t-value	P-value	BMI
Anti HEV IgG	Positive (n=141)	70.07 ± 41.05 U/L	1.20	0.23	28.7 Kg/m^2^
	Negative (n=54)	68.48 ± 31.64 U/L			30.9 Kg/m^2^
	Total = 195				
Anti HEV IgM	Positive (n=7)	110.85 ± 129.97 U/L	1.97	0.02	27.5 Kg/m^2^
	Negative (n=188)	68.09 ± 30.6 U/L			28.8 Kg/m^2^
	Total = 195				

**Table-II:** Age-wise Hepatitis E IgM seroprevalence.

Sr No	Age Group	Anti HEV IgM (n=515)		Seroprevalence
		Negative (n=484, 94%)	Positive (n=31, 6%)	
1.	18-27	200 (41.3%)	20 (64.5%)	9.09 %
2.	28-37	199 (41.1%)	8 (25.8%)	3.86 %
3.	38-47	74 (15.3%)	3 (9.7%)	3.89 %
4.	48-57	11 (2.3%)	0 (0)	Nil

* Percentages are based on the total study population (n = 515).

**Table-III:** Age-wise Hepatitis E IgG seroprevalence.

Age Group	Anti HEV IgG (n=515)		Seroprevalence
	Negative (n=149, 28.9%)	Positive (n=366, 71.1%)	
18-27	72 (48.3%)	148 (40.4%)	67.27 %
28-37	57 (38.3%)	150 (41.0%)	72.46 %
38-47	18 (12.1%)	59 (16.1%)	76.62 %
48-57	2 (1.3%)	9 (2.5%)	81.81%

* Percentages in parentheses represent proportions of the total study population (n = 515).

## DISCUSSION

In this prospective study, involving 515 healthy blood donors, it was observed that a high seroprevalence of anti-HEV IgG (71.1%) and anti-HEV IgM (6%) were found, it indicates that both the past exposure and ongoing transmission of Hepatitis E virus exist in this population. Among the younger donors (aged 18-27 years), highest IgM positivity was found, which suggests recent infection trends in younger adults. A substantial correlation between HEV IgM positivity and elevated ALT levels was noted, while no HEV RNA was detected in any collected sample, most likely due to low viral load below the detection threshold. The mean BMI (overall) of the participants was >25 kg/m², showing a tendency towards overweight status, which may have clinical associations in liver function and disease progression.

Hepatitis E is a disease that affects people all over the world. Although, HEV is self-limiting but can lead to severe consequences among pregnant or immunocompromised and chronic liver disease population. Literature reports HEV incidence to be associated with antenatal maternal mortality and significant neonatal illness. Mothers infected with HEV are more susceptible to have stillbirths and fetal complications.^15^,^16^

HEV is mainly associated with poor sanitation and hygiene habits however blood transfusion is also an important transmission route behind the increased prevalence of HEV.^17^ This is the major reason that has outdistanced the theory for higher prevalence of HEV in countries with low socioeconomic conditions^17^ with no impact of socioeconomic status and incidence of HEV.

Blood is essentially screened to determine the prevalence of transfusion transmitted infection in healthy individuals as well as to guarantee the security of the blood and blood product supply. But still high incidence of anti-HEV IgG among blood donation volunteers has also been observed.^18^ This may be because majority of blood donors in Pakistan are among those who have not previously donated blood.^19^,^20^ which can be suggestive of obliviousness of the infection among this population. However, other research suggest that blood donors could not accurately represent the overall population since their varied traits could cause the incidence rate to be either overstated or underestimated.^21^

Many European nations have implemented routine HEV testing or targeted screening of blood donated by high-risk patients to prevent viral transmission by transfusion as a result of the observation of high incidence of HEV viraemia in blood donors.^22^ According to earlier research, Asia has the highest regional anti-HEV-IgG seroprevalence which aligns with our study with high prevalence observed. 23

According to a review conducted by Belei et al.,^23^ higher seroprevalence of hepatitis E infection was observed in younger age group (18-27 years) compared to middle aged population combined (age 28-47 years).^23^ This also shows an inverse correlation with Indian studies of Gajjar et al.^24^ and Farooqi et al.^25^ in which older population had a higher prevalence of Hepatitis E. Higher seroprevalence of IgM in younger population was found in our study which may be due to susceptibility pattern of HEV which primarily affects young adults.^25^-^27^ In our study, we also found positive correlation of IgM incidence with high levels of ALT which is also supported by the study published by Al Absi and colleagues in aspect of population of South East Asia.^27^

In our study, even though we found positive seroprevalence of HEV but did not observe any HEV RNA positive sample which might be due to the fact of less number of copies which may be below the minimum detectable range of our kit Nonetheless, similar observation was also reported by Costa and colleagues.^28^

Our study also revealed an increased average BMI of the total study population (i.e., >25 kg/m^2^). As research by Fan et al concluded the association of raised BMI with progression towards nonalcoholic fatty liver disease and NAFLD is linked to male patients and hepatitis E.^29^-^32^ Therefore, we can presume that our study population is towards higher risk for developing NAFLD. Further large-scale studies are needed to establish confirmed correlation between HEV with raised ALT levels and BMI levels. Additional studies related to raise Hepatitis E prevalence among younger population in Pakistan are required.

### Limitations:

Our study included only male participants which is due to the fact that in Pakistani population, majority of blood donors are male which relates to the norms of our population and society^33^ and also because of prevalence of Iron deficiency in females. Detection limit of 500 copies of the Viral Extraction kit could have missed the individuals with resolving or early hepatitis with low viral load, hence using better sensitivity kits is suggested.

## CONCLUSION

High seroprevalence of Anti HEV IgM among healthy blood donors emphasizes the need for robust measures of routine screening for HEV of all blood products in endemic countries to prevent transfusion dependent transmission with special consideration when the recipients are either immunocompromised or pregnant.

### Author`s Contribution:

**LK:** Conceive the idea, analysis of data, final editing critical review of manuscript and responsible for accuracy of study.

**AR:** Writing of proposal, data collection, statistical analysis and writing of first draft.

**SA:** Designing of the study, Data Acquisition and analysis, second revision of manuscript.
